# Dental Amalgams

**Published:** 1896-11

**Authors:** J. M. Walker


### MONTHLY DIGEST.

Prepared for the DENTAL REGISTER by MRS. J. M. WALKER.

### DENTAL AMALGAMS.*

DR. A. C. HEWITT, in an article on the "Uses and Abuses of Amalgam," attributes failure, in many cases, not to the alloy itself, but to the "lazy, shiftless, dishonorable and dishonest workmanship" as contrasted with the daily and yearly work of honorable men without whose work amalgam could not have attained the rank it now securely holds.

Among the abuses pointed out are, failure to use efficient means of securing perfect dryness, carelessness in divesting the alloys of mercurial super-saturation, failure to so pack as to secure equal density throughout the mass, exposing the comminuted material to the oxidizing influences of air and light, instead of keeping it in bars or ingots to be filled or turned to ribbons or chips as required for immediate use, mixing the alloy and mercury with soiled fingers in sweaty palms, thereby increasing the porosity of the amalgam and lessening its capacity to bar out oral fluids, etc., etc.

When either purse or tooth demands something else than gold, whatever material is used, skill and honest endeavor are always demanded on the part of the operator. Dr. Hewitt believes, from experience and observation, that when an amalgam filling is indicated it should be both a stopping and a remedial agent, and he, therfore, prefers copper amalgam as having both tonic and stimulating properties, devoid of escharotic effects.

He reviews the objections urged against copper amalgam, viz.; Cupping or washing out on occlusal surfaces, darkening tooth substance, staining contiguous gold work, etc., and the methods of overcoming these objectionable features, which, he claimed, required only a correct formula, and the observance of chemical and metallurgical laws, uniformity of methods giving uniform results. He then gives, in detail, the methods by which he secures the desired results, the method of preparing the cavity, of preparing the amalgam, and of inserting the alloy, the presence of moisture being the worst for and abuse of copper amalgam.

Dr. E. D. Downs, in a paper on "The Choice of Filling Materials," read before the Union Convention, at Buffalo, considers the use of filling materials, other than gold, simply as "deviations" from the standard, governed by a variety of considerations as, the skill of the operator, the health of operator or patient, the purse of the patient, the conditions presenting in the mouth, the quality of the teeth, etc., etc.

He would use gold under the age of sixteen years only in exceptional cases, preferring gutta-percha in approximal surfaces, in crown, oxyphosphate and occasionally, amalgam, in crown cavities. He considers amalgam our sheet-anchor where everything else fails. If a patient can not afford gold in the back teeth, then, put into carefully-prepared cavities, carefully-inserted and well-filled amalgam fillings, and thank God you are a good dentist, and a benefactor to suffering humanity—a greater honor to the profession than one who would turn him from the door to fall a victim to the hypodermic needle.

